# On the Uses of Turpentine

**Published:** 1854-05

**Authors:** Samuel Thompson

**Affiliations:** Albion, Illinois


### THE NORTH-WESTERN

### MEDICAL AND SURGICAL JOURNAL.

### NEW SERIES.

VOL. III. MAY, 1854. NO. 5.

### ORIGINAL COMMUNICATIONS.

*Transactions of the Illinois State Medical Society, for the year* 1853. "On the uses of Turpentine; by SAMUEL THOMPSON, M. D., of Albion, Illinois."

*A Dictionary of Practical Medicine*, Comprising General Pathology, the Nature and Treatment of Diseases, Morbid Strictures, &c. By JAMES COPLAND. M. D., F. R. S. Re-published by Harper & Brothers, New York.

"Render unto Cæsar the things that are Cæsars."

(*Continued from page* 54.)

We have no doubt that Dr. Thompson will find by a further experience of the use of Turpentine in Diarrhœa alba, at least in that form which arises from an excited, inflamed or ulcerated condition of the mucous membrane, especially when the disease is of some duration, and is passing or has passed into a chronic state—that it is, without any exception, the best remedy that we possess for removing the morbid condition on which the diarrhœa depends, if one medicine only is used—although in a majority of cases, other medicines may be given with advantage, either separately or in combination with it.

We will here relate a case, to which we were called early in the morning of July 12th, 1844, and in which Turpentine was used alone in the beginning, but unsparingly and successfully. The patient was a girl three years and two months old. We had heard of the case some ten days before we saw it, and almost daily after—and that it was gradually getting worse, and at two o'clock in the morning had been told that the child was dying. We had barely reached home when the father rode up, and asked if we would go over to Wyoming and see his child, which he had no doubt was dying. He told us that the senior Dr. had not seen it for three days—failing to come yesterday, he presumed the Dr. had given up the case. The Dr. in attendance had refused to give it any medicine, or do anything else for it since the day before, for the child was dying, and it would be useless and cruel to disturb it. Without any real occasion, there had been some predudice in us towards this family, and we were perfect strangers to every member of it; this feeling was known. for we always take the trouble to let any one whom we dislike know the fact, for to us it is the most unpleasant task belonging to the profession, to attend such in any case of sickness, and especially if the sickness be of a dangerous or fatal character, and this was the reason why we did not earlier see the case.

On arriving at the Garden gate we were met by the mother, who convulsively took our arm, and said with an evident depth of feeling, that mothers only experience, "Dr. H., my only child is dying, and I want you to do something for it, although you may know that it will do it no good, for it will relieve my heart which is breaking."

On entering the room we found the child in a cot, breathing rapidly, and with a death-like countenance: a napkin had just been removed, and the discharge contained nothing except mucus and muco-purulent matter. We stayed not to make inquiries—"desperate cases, require desperate remedies," and the sooner they are used the better. We gave the child two tea-spoonfuls of Turpentine, losing something like a third in the exhibition of it. An ounce was mixed with three ounces of gruel and thrown up the bowels, and a flannel moistened with Turpentine was wrapped round the body and covered with a greased newspaper; this occupied about five minutes. We then retired for a few minutes, and on our return ventured, from the great change in the countenance, to relieve the breaking heart by giving, somewhat gradually, however, a favorable prognosis.

This is another case where the system immediately recognized the efficiency of the medicine.

We then took breakfast, which occupied some fifteen minutes, and on our return to the patient found it breathing easily and sleeping sweetly, although the surface was literally as "red as a boiled lobster." There was no farther difficulty; a few drops of Turpentine three times a day for four days arrested the mucous and muco-purulent discharges, and a few doses of Hydrarg. cum Creta occasionally given for five days, completed the cure; the discharges when they became feculent manifesting an absence of bile.

We are totally ignorant of the prior treatment of the case, although the Physician in attendance was present when we entered; we had not spoken to him for over two years, and the feelings we had towards him forbade any enquiry.

There is another form of Diarrhœa in which the discharges are *white*, large in the beginning of the disorder, contain a great quantity of gas in small vesicles, and in appearance very much resemble yeast. This form is generally met with in children during the hot summer months, and probably depends on a torpid condition of the liver, and it readily yields to the Hydrarg. cum creta given three times a day until the stools become yellow, and once every, or every other day for a week, or longer should the discharges show a tendency to return to their former condition.

And yet there is another form of Diarrhœa in which the discharges are white, but less feculent, more offensive and more frequently voided than in the preceding variety. We have met with it more frequently in the spring than at any other season of the year—the abdomen is always large and the countenance sallow and unhealthy. For this variety we have found the Iodide of Mercury, given until the discharges become yellow, and afterward the Sulphates of Quinine and Iron, the best treatment.

We refer to these, because Turpentine in our experience has been useless as a remedy in such cases, and yet they would evidently come under the somewhat vague designation of white Diarrhœa; but on the other hand, in Diarrhœa arising from the pathological conditions first mentioned, it is by far the most efficient remedy we possess, and we say this on an experience of over twenty years and in hundreds of cases.

We have only had an opportunity of giving Turpentine in one case of hœmatemesis, and although no vomiting of blood occurred after its exhibition, still we had some little doubt whether its arrest depended upon that medicine; but the authority of Copland is warmly in its favor and to the point.

"Notwithstanding its usual nauseating effect, Turpentine is generally retained in hœmatemesis, and it allays the vomiting by arresting the hamorrhage. It may be given in any dose, from twenty to thirty drops every half hour, to half an ounce or more at considerable intervals; it may also be administered in *enemata*, or applied externally in the form of *Liniment*, or *epithem*. I have resorted to this practice upwards of twenty years, and am convinced that it is safer and more appropriate, generally, than any other yet recommended."—*Copland, op. cit. Volume* 2, *Page* 98.

